# Costameres, dense plaques and podosomes: the cell matrix adhesions in cardiovascular mechanosensing

**DOI:** 10.1007/s10974-019-09529-7

**Published:** 2019-06-18

**Authors:** Brian Sit, Daniel Gutmann, Thomas Iskratsch

**Affiliations:** Division of Bioengineering, School of Engineering and Materials Science & Institute for Bioengineering, Queen Mary University of London, London, UK

**Keywords:** Mechanosensing, Cardiomyocytes, Smooth muscle cells, Costameres, Dense plaques, Podosomes

## Abstract

The stiffness of the cardiovascular environment changes during ageing and in disease and contributes to disease incidence and progression. For instance, increased arterial stiffness can lead to atherosclerosis, while stiffening of the heart due to fibrosis can increase the chances of heart failure. Cells can sense the stiffness of the extracellular matrix through integrin adhesions and other mechanosensitive structures and in response to this initiate mechanosignalling pathways that ultimately change the cellular behaviour. Over the past decades, interest in mechanobiology has steadily increased and with this also our understanding of the molecular basis of mechanosensing and transduction. However, much of our knowledge about the mechanisms is derived from studies investigating focal adhesions in non-muscle cells, which are distinct in several regards from the cell–matrix adhesions in cardiomyocytes (costameres) or vascular smooth muscle cells (dense plaques or podosomes). Therefore, we will look here first at the evidence for mechanical sensing in the cardiovascular system, before comparing the different cytoskeletal arrangements and adhesion sites in cardiomyocytes and vascular smooth muscle cells and what is known about mechanical sensing through the various structures.

## Introduction (i.e. why care about mechanosensing in the cardiovascular system?)

Cardiovascular diseases (CVD), including coronary artery disease, myocardial infarctions (MI), hypertrophic cardiomyopathy (HCM) and dilated cardiomyopathy (DCM) are the primary cause of mortality worldwide and a huge economic burden. CVD costs the US both, directly and indirectly, $320 billion per year, with this figure set to rise to $918 billion by 2030 (Travers et al. 2016). Similarly, in the EU, CVD costs €169 billion per year, equating to 62% of their healthcare costs. This is contributed to by deaths as well as the long-term impact of a reduced quality of life and life expectancies (Leal et al. 2006). Clinically, all these conditions have in common a stiffening of the extracellular matrix (ECM) through fibrosis and it has become clear that this stiffening is a key contributing factor in the progression of the CVD. The cells in the heart or vessel walls sense the changes to chemical and mechanical signalling and respond to the restructuring of the ECM by changing their phenotype; e.g. cardiomyocytes become less contractile due to changes in gene expression and myofibrillar organisation (Ward and Iskratsch 2019), while vascular smooth muscle cells can switch from a contractile to a synthetic phenotype that allows them to start migrating or invading into the inner layer of the vessel wall (Hartman et al. 2016; McDaniel et al. 2007).

Our knowledge about mechanosensing is mostly derived from migratory cells e.g. in cancer, while information about mechanosensing through specific cardiomyocyte or vascular smooth muscle adhesions is lacking. A PubMed keyword search for “mechanotransduction” and “Focal Adhesion” for instance yields 467 papers, while the combination with “Costamere” or with “Podosome” comes up with only ten results each. Of course, this keyword search is not showing the complete picture. Nonetheless, it demonstrates a certain focus of the mechanobiology field on motile cells e.g. in cancer where focal adhesions are the primary mechanosensitive structures. Here we want to disseminate the similarities and differences between the different integrin adhesion structures. For this, we will first look at the changes to the ECM composition and stiffness in the heart and the vessel wall respectively, before discussing the different cell–matrix adhesions in cardiac and VSMCs and their involvement in mechanical sensing.

## Structure of the cardiac ECM and mechanical changes in heart disease

In the heart, the ECM provides physical support and facilitates cellular functions (Chang et al. 2016). It helps to connect and organise the myocytes, absorb and transmit forces generated on the tissue and thereby prevent damage during systole and diastole (Weber et al. 1994). Cardiac fibroblasts produce the majority of the cardiac ECM. The cardiac collagen fibrils are mainly comprised of collagens type I and III. Collagen type IV sits in the basement membrane which surrounds the myocytes (Brown et al. 2005; Lockhart et al. 2011; Rienks et al. 2014). Other constituents of the ECM include glycoproteins (e.g. fibronectin and laminin), proteoglycans (especially heparan sulfate proteoglycans) and elastin. These molecules interact with cell surface receptors, such as integrins which link the ECM to the internal cytoskeletal network and function also as mechanical sensing and signalling hubs (Awada et al. 2016; Baudino et al. 2006; Ward and Iskratsch 2019). However, the composition of the ECM is dynamic and changes during development and in disease. Fibronectin and collagen are important binding partners in the developing heart, but laminin is up-regulated, becoming the main integrin binding partner in the adult heart (Marijianowski et al. 1994; Oliviero et al. 2000; Terracio et al. 1991).

Diseases or injuries, such as MI damage the myocardium, with ischaemia triggering an inflammatory response, changing the molecular composition and cellular morphology. The abnormal ECM constituents change the functionality of the heart (Awada et al. 2016; Sullivan et al. 2014). After an MI, the death of cardiomyocytes en masse triggers a cascade resulting in fibrotic tissue replacing the dead cells. Inflammation activates immune pathways and oxidative stress, leading to the mobilisation of toll-like receptors which activates NF-κB, resulting in a further inflammatory pathway upregulation. The inflammatory cytokines (TNF-α, IL-1β and IL-6) cause cellular apoptosis and fibrotic tissue formation (Bujak et al. 2009). The effects of NF-κB can be protective, however, prolonged presence induces a damaging inflammatory response. Inflammatory cytokines attract lymphocytes and macrophages to the site, removing dead cardiomyocytes. Matrix Metalloproteinases (MMPs) are activated, degrading the ECM, causing the accumulation of collagen particles (Etoh et al. 2001). This activates neutrophils, further enhancing the inflammatory response. MMPs, ROS and the acidic environment of damaged tissue activate TGF-β1 release, which stimulates inflammatory cells, enhancing fibrotic remodelling (Kong et al. 2014). The remodelling itself includes the activation of the CFs, secretion of the stiffer collagen I versus III, as well as fibronectin and importantly also lysyl oxidases that lead to increased cross-linking of the ECM (Hermida et al. 2009; Hughes et al. 2008; Maki 2009; Marijianowski et al. 1994; McCormick et al. 1994; McCormick and Thomas 1998). Overall, these changes result in an increased stiffness of the fibrotic myocardium, from approx. 10 kPa in the healthy to > 50 kPa in the infarcted or fibrotic tissue although there is currently still a large variability depending on the model system and techniques used to measure the stiffness (Ward and Iskratsch 2019).

## Structure of the vessel wall ECM and disease associated mechanical changes

Changes to the vessel structure, associated with ageing and atherosclerosis are dramatically increasing the risk of CVD. These structural changes lead to altered topography as well as enhanced stiffness (Gozna et al. 1973; Lakatta et al. 1987; Laurent et al. 2001). Because of this, macroscale analysis of vascular stiffness, such as pulse wave velocity is used to predict the risk for cardiovascular disease. Arteries are composite structures (Wagenseil and Mecham 2009). The intimal layer contains basement membrane and endothelial cells. It is separated from the media by an internal elastic lamina, containing longitudinal elastin fibres. The medial layer is the largest contributor to the elastic properties of the arterial wall. It is populated by smooth muscle cells and contains collagen and circumferential elastin fibres. The outer adventitia layer contains fibroblasts and a looser collagen and elastin matrix. Owing to increased association of VSMCs with collagen under pressure, arteries display non-linear stress–strain patterns (i.e. the elastic modulus increases when they are stretched through blood flow) (Bank et al. 1996; Wagenseil and Mecham 2009; Zhou and Fung 1997). The measured moduli differ between the various arteries and e.g. pulmonary arteries are stiffer than the aorta and further the descending thoracic aortic tissue is stiffer than ascending aortic tissue (de Beaufort et al. 2018; Grant and Twigg 2013). The measurements further vary between longitudinal and circumferential strain. Moreover, Young’s moduli are sometimes separately measured for elastic lamellae, elastin-collagen fibers and collagen fibers (Khamdaeng et al. 2012). Finally, the mechanical properties differ between macro and microscale (Kohn et al. 2015). At the microscale, elastic moduli between 20 and 70 kPa have been measured with nanoindentation or micropipette aspiration assays (Hanna et al. 2014; Hemmasizadeh et al. 2012; Matsumoto and Hayashi 1996), however, no large-scale study of changes during ageing have been performed. At the macroscale (circumferential and longitudinal) moduli from 100 kPa to several MPa have been measured with a strong trend from low stiffness in childhood to high values in old age and diseases, including atherosclerosis or in patients with aneurisms (Khanafer et al. 2011). These changes are largely coming from fragmentation, calcification and degradation of elastin fibres, shifting the load increasingly onto stiffer collagen fibrils (Kohn et al. 2015; Schlatmann and Becker 1977). Additionally, collagen expression and crosslinking by non-enzymatic glycation increases with age, further stiffening the vessel walls (Schlatmann and Becker 1977; Schleicher et al. 1997; Sims et al. 1996).

## Evidence for rigidity sensing of cardiac and smooth muscle cells

It is well established that many cell types, including cardiomyocytes and vascular smooth muscle cells, can sense the changes in the ECM stiffness, through changes in the transmission of mechanical forces, altering their behavioural response accordingly (Iskratsch et al. 2014; Roca-Cusachs et al. 2012). Cell area, contraction frequency, percentage of contractile (isolated) cells, force and contractile work of primary or stem cell derived cardiomyocytes change in response to matrix stiffness (Engler et al. 2008; Hazeltine et al. 2012; Hersch et al. 2013; Li et al. 2016; Pandey et al. 2018; Ribeiro et al. 2015; Rodriguez et al. 2011, 2014). Generally the studies found that contractile force increases with stiffness (Hazeltine et al. 2012; Hersch et al. 2013; Rodriguez et al. 2011), however when micropatterning was used to restrict the cell area and shape there was a peak in contractile force at 10 kPa, while at higher stiffnesses myofibrillar rupture led to reduced mechanical forces (Ribeiro et al. 2015). Also, contractile work was found to be highest at 10 kPa corresponding to the native tissue stiffness (Engler et al. 2008). From a signalling pathway perspective cardiomyocytes respond to the stiffness by changing expression of cardiac developmental agonists such as PI3K/AKT or p38/JNK pathway components, or antagonists (e.g. canonical Wnt signalling components), whereby differences exist between cells that are exposed to a static stiffness or a hydrogel that becomes stiffer over time (i.e. mimicking the changing stiffness during heart development)(Young et al. 2014). Also lipid pathways (Li et al. 2016) calcium handling (Rodriguez et al. 2011; van Deel et al. 2017) and possibly also nitric oxide signalling (Jian et al. 2014) are affected by the stiffness and presumably involved in the mechanosignalling.

Vascular smooth muscle cells similarly respond to a range of physical/mechanical cues including pressure, stretch, topography and stiffness (Chaterji et al. 2014; Kim et al. 2015; Le et al. 2018b; McDaniel et al. 2007). Matrix stiffness (and stiffness gradient) can induce a phenotypic switch of VSMC and affects VSMC stress fibre organisation, contractility or migration rate (Isenberg et al. 2009; Peyton et al. 2008; Peyton and Putnam 2005; Sugita et al. 2019), although effects in 3D were less pronounced than in 2D (Peyton et al. 2008). Stiffness further regulated VSMC cell cycle and proliferation (Bae et al. 2014; Klein et al. 2009). Furthermore, VSMC can form podosomes at soft 3D matrices without chemical stimuli (Furmaniak-Kazmierczak et al. 2007). Podosomes are protrusive structures at the matrix interface of the plasma membrane. They are a critical element of the phenotypic switch from a contractile to a synthetic phenotype (Hartman et al. 2016; McDaniel et al. 2007) and regulate matrix adhesion and matrix degradation through the targeted secretion of matrix metalloproteinases (MMPs) (Vijayakumar et al. 2015).

## Mechanical sensing through cell–matrix adhesions: common themes and differences between cell types

A comparison of proteomic studies of the adhesion complexes from various cell types resulted in a 2412-protein integrin adhesome (Horton et al. 2015). However only 60 of these were present in at least five data sets, indicating a large variability between cell types and conditions. Approximately half of the consensus adhesome was matching a literature curated adhesome, while the rest was previously less well characterized (Winograd-Katz et al. 2014).

This suggests that although there are important differences in the composition (as well as the organisation of the attached cytoskeleton and the force transmission from the cytoskeleton), key proteins are shared between different cell types and presumably also adhesion structures in cardiomyocytes and smooth muscle cells; indeed many focal adhesion proteins have been initially isolated from smooth muscle such as chicken gizzard (Burridge and Connell 1983; Feramisco and Burridge 1980; Geiger 1979; Kelly et al. 1987).

In all cell-ECM structures integrins connect to the ECM and span through the membrane. At the cytoplasmic tails are a range of integrin binding proteins, including kindlin, paxillin, or FAK, as well as actin binding proteins (e.g. vinculin or VASP), or proteins that bind both integrin and actin (e.g. talin, α-actinin, filamin) (Winograd-Katz et al. 2014). These complexes connect the integrins to the cytoskeleton and play signalling roles. Integrins are activated either outside-in through binding of an ECM ligand, or inside-out through binding of kindlin and talin that stabilize the open conformation and promote ECM ligand binding, while Filamin on the other hand inhibits integrin activity (Liu et al. 2015). Talin binds to integrins through its head domain and has two actin binding sites on the rod domain (and also a less-well characterized actin binding site on the head) which connect then to the actin cytoskeleton (Klapholz and Brown 2017; Legate and Fassler 2009). If force is applied through the cytoskeleton (either through myosin contractions or actin assembly) this can lead to stretching of talin and opening of cryptic vinculin binding sites on stiff environments, while the force will be transmitted through the entire complex onto the matrix and deform the matrix on soft environments (Roca-Cusachs et al. 2012). Vinculin then reinforces the connection by binding to both talin and actin. Nevertheless, talin’s connection to actin is not continuous but rather it cyclically engages and disengages in what has been termed a stick–slip mechanism, leading to repeated extension of talin (Margadant et al. 2011). Similarly, even though some integrins can form catch bonds and therefore integrin-ECM bond lifetimes increase under force (Roca-Cusachs et al. 2012), they are still only in the order of seconds (Kong et al. 2009), thus making focal adhesions dynamic structures. Due to the repeated engagement and disengagement the cell-ECM adhesion is frequently compared to a molecular clutch (Elosegui-Artola et al. 2018; Mitchison and Kirschner 1988; Swaminathan and Waterman 2016). An updated model of the molecular adhesion clutch suggests a load and fail cycle wherein the force initially builds up resulting in talin stretching and recruitment of vinculin and adhesion reinforcement. Eventually the force build-up leads however to bond destabilisation, and clutch disengagement as well as release of all forces in a catastrophic event. On soft substrates, the rate of load transmission is below the integrin–ECM bond lifetime and the bond dissociates before talin can unfold. Above the optimal rigidity, force builds up too fast for additional clutches to stabilize the adhesion and therefore it disengages (Elosegui-Artola et al. 2016, 2018; Swaminathan and Waterman 2016). Integrins are structurally focal adhesions are elongated and consist of multiple subunits that are about 300 nm in diameter, each attached to a single stress fibre (Hu et al. 2015). The components are stratified into multiple layers that have been termed ‘integrin signalling’ (containing e.g. integrin cytoplasmic tails and talin head domains), ‘force transduction’ (containing e.g. talin rod domain and vinculin) and ‘actin regulatory’ layer (containing e.g. VASP) (Kanchanawong et al. 2010).

### What are the differences?

But as demonstrated by the comparison of the proteomic analysis data, focal adhesions differ between cell types and even within a single cell depending on space and time (Horton et al. 2015). During adhesion formation early integrin clusters are connected to small contractile units (composed of non-muscle isoforms of muscle sarcomeric proteins, such as actin, myosin, α-actinin, tropomyosin, tropomodulin, the formin FHOD1, etc.) that apply force onto integrins and are involved in rigidity sensing (Iskratsch et al. 2013; Meacci et al. 2016; Wolfenson et al. 2016). In contrast, (mature) peripheral adhesions are connected to non-contractile dorsal stress fibres while central adhesions are attached to contractile ventral stress fibres (Tojkander et al. 2012). Contractile force to the peripheral adhesions is indirectly provided through transverse arcs that connect to the dorsal stress fibres, but is not sufficient for adhesion growth and mechanosensing, which additionally depends on the force provided through ARP2/3 dependent actin polymerization (Oakes et al. 2012, 2018; Pasapera et al. 2010). However tension on talin is higher in peripheral versus central adhesions, further suggesting that these are differently organised (Kumar et al. 2016).

To complicate matters further, even peripheral focal adhesions are not all the same and can be divided into (mechanically) stable or tugging focal adhesions, whereby the latter are exposed to fluctuations in the level of force and the position within the adhesion where the force is applied (Plotnikov et al. 2012); only the latter is required for mechanical sensing and e.g. durotaxis, i.e. the migration guided by a stiffness gradient.

**Fig. 1:**
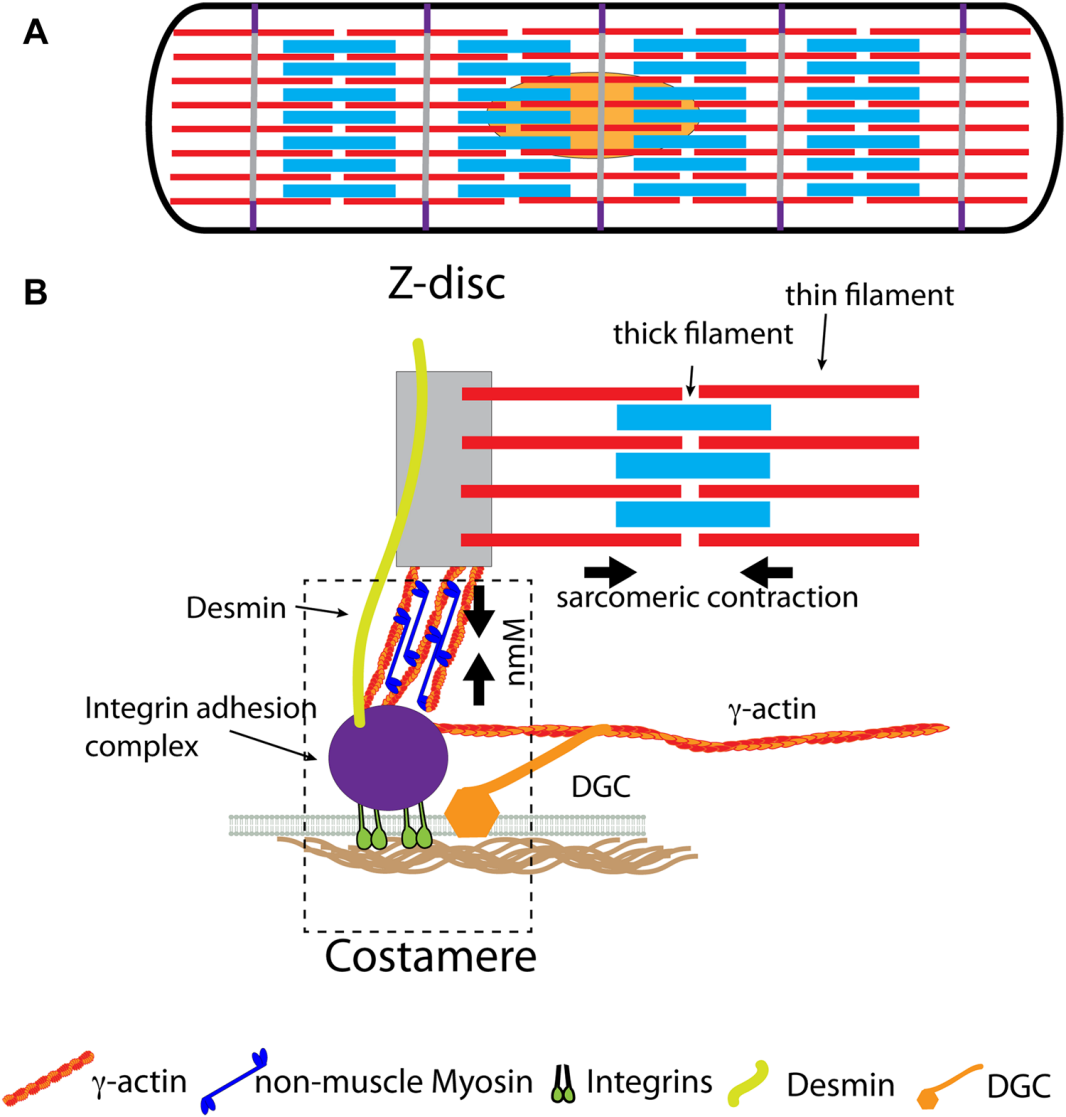
The cardiomyocyte costamere. The sarcomeres are connected to the ECM through integrins, the dystrophin-glycoprotein complex (DGC) and desmin. **a** Side view of cardiomyocyte with thin filaments in red and thick filaments in light blue as well as Z-discs in grey and costamer in purple. **b** Side view of a costamere with the adhesion complex (i.e. talin, vinculin and other adaptor as well as signalling molecules) in purple, the DGC in orange and the desmin intermediate filaments in lime green. Arrows indicate the contractile forces from myofibrillar and non-myofibrillar structures. *nmM* non-muscle myosin. (Color figure online)

**Fig. 2:**
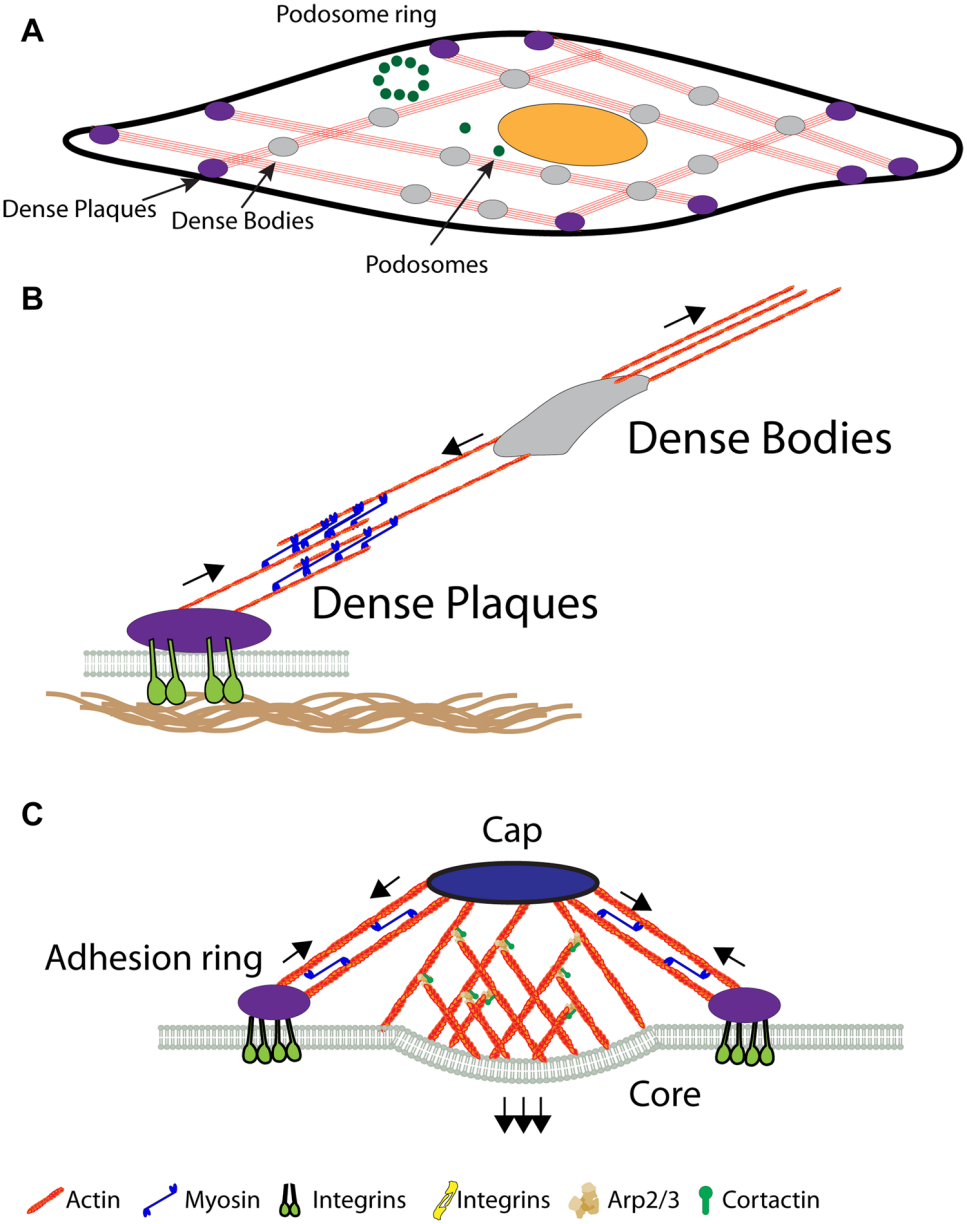
Vascular smooth muscle cell adhesions. **a** Top view of a VSCM, with dense bodies in grey, dense plaques in purple and podosomes in green. **b** Side view of dense plaques attached to contractile stress fibres and dense bodies in the cytoplasm. Arrows indicate contractile forces of the stress fibres. **c** Side view of a podosome. The forces of the protrusive core with branched actin, as well as the tensile force from the adhesion ring are indicated by arrows. (Color figure online)

Further, the different cell types vary in their organisation widely and this includes also different cytoskeletal organisation and cell–matrix interaction sites through which the cells can sense the mechanical stimuli (Figs. 1 & 2). Because mechanical sensing is depending both on the organisation of the (contractile force-producing) cytoskeleton as well as the cell–matrix adhesion sites, we next look at the different cytoskeletal arrangements and adhesion sites in cardiomyocytes and vascular smooth muscle cells and then compare what is known about the regulation of mechanical sensing.

### Costameres

Costameres, the main matrix attachment sites in cardiomyocytes connect the cytoskeleton to the ECM not only through integrins and associated proteins, but also through the dystrophin-glycoprotein complex (DGC) (Fig. 1). Additionally, the costameres connect to the myofibrils through the intermediate filament protein desmin, whereby all three components appear to be involved in mechanical sensing and signal transduction (Ward and Iskratsch 2019). The integrin adhesion component has many of the proteins that are also found in focal adhesions, including talin and vinculin which attach to cytoplasmic γ-actin that is further connected to the sarcomeric Z-disc through actin crosslinkers such as α-actinin and plectin (Ervasti 2003). The attachment of the sarcomeres to the costameres through cytoplasmic actin leads to a situation where the forces from the regular sarcomeric contractions can be modified through non-muscle myosin, which contracts the cytoplasmic actin (Fig. 1) (Pandey et al. 2018). Moreover, non-muscle myosin is localized at the costameres especially in heart disease, suggesting that this modulation can lead to an alteration of mechanical sensing with potentially adverse effects on the disease progression (Pandey et al. 2018). Together the forces are sensed at the adhesions where it leads to different dynamics of talin stretching, depending on the stiffness of the ECM. Because talin has a large range of binding partners and all the rod domains can unfold and refold under force, such differences in stretching dynamics are expected to alter mechanical signal transduction beyond vinculin binding and adhesion reinforcement and indeed force dependent talin binding has been reported already for other proteins than vinculin (Haining et al. 2018; Klapholz and Brown 2017; Yao et al. 2016). In addition to talin the costameres contain a number of proteins that are general mechanosensors and included in the consensus adhesome (e.g. ILK-PINCH-Parvin) (Jani and Schock 2009; Li et al. 2012) as well as muscle specific proteins such as MLP (Flick and Konieczny 2000; Knoll et al. 2002). Importantly also, the isoforms of integrins and several adapter proteins are different in cardiomyocytes compared to many non-muscle cells (β1D vs β1A integrin, talin 2 vs talin 1) which affects binding affinities, dynamics and signaling (Hawkes et al. 2019; Ward and Iskratsch 2019). E.g. a reduced binding of kindlin and paxillin to β1D was reported, suggesting that talin binding might be the main activator of β1D integrin in muscle (Soto-Ribeiro et al. 2019; Yates et al. 2012). Moreover many isoforms switch back to embryonic splice variants in cardiac disease and thereby again modifying affinities and potentially other binding partners (Ward and Iskratsch 2019).

The intermediate filament protein desmin is flexible and seems to serve a function as load bearing spring, i.e. to absorb contractile forces between Z-disc, microtubules and ECM (Hein et al. 2000; Robison et al. 2016). Abnormal desmin levels and/or filament organisation are linked to heart disease presumably due to the lack of this force buffering capability (Bouvet et al. 2016; Clemen et al. 2015; Geisler and Weber 1988; Thornell et al. 1997).

The dystrophin glycoprotein complex (DGC) seems to serve a similar function as shock absorber (Le et al. 2018a). It consists of dystrophin, the transmembrane dystroglycan and sarcoglycan-sarcospan subcomplexes as well as the subsarcolemmal proteins dystrobrevins and syntrophins. Dystrophin binds to actin through its N-terminal and rod domain and to dystroglycan through the cysteine-rich C-terminal domain, while dystroglycan connects to laminin in the basement membrane (Lapidos et al. 2004). In the heart dystrophin is detected all along the membrane, albeit more concentrated at the costamere (Kawada et al. 2003; Stevenson et al. 1997). Dystrophin has roughly equal affinities to sarcomeric α-actin, as well as β- and γ-actin (Renley et al. 1998). However, it appears from immuno-electronmicrographs that it is mostly oriented near-parallel to the cytoplasmic surface, with only a few dystrophin molecules connecting to the farthest out myofibrillar actin filaments (Wakayama et al. 1993). Thus, it is assumed that it primarily interacts with subsarcolemmal γ-actin (Fig. 1) and through this interaction helps to protect sarcolemma from fragmentation or micro-rupturing and mitochondria from deformation during the myofibrillar contraction (Kawada et al. 2003). Therefore, absence of dystrophin, or other members of the DGC can lead to a progression of the dystrophic phenotype in hearts during aging, which is accelerated through physical exercise (Nakamura et al. 2002). Apart from this mechanical role the DGC is also a signalling hub and involved in ion transport regulation, nitric oxide, MAPK or Rac1 GTPase signalling [reviewed in (Constantin 2014)]. Therefore, some of these signalling pathways might be also regulated through the changing mechanical environments, or lack of buffering of the force in absence of the DGC.

### Dense plaques

Historically dense plaques were defined by their appearance as densely stained structures in electron micrographs of smooth muscle cells (North et al. 1993). Not to be confused with cadherin-based cell–cell adherens junctions (Dorland and Huveneers 2017), or intermediate filament-based “dense bodies” (Tang 2008), dense plaques here are integrin-rich adhesion sites of smooth muscle cells with associated actin networks (Morgan et al. 2007; Turner et al. 1991; Zhang and Gunst 2008), and are responsible for force transmission among smooth muscle cells across the ECM (Gunst and Tang 2000) (Fig. 2a, b). Dense plaques again contain the same repertoire of molecules that are also present in focal adhesions, including integrins, α-actinin, talin, vinculin or filamin (many of which in fact were first identified in smooth muscle as discussed above) (Draeger et al. 1990; Turner et al. 1991), but additionally there are also components that are specific for smooth muscle, including the LIM domain protein LPP or metavinculin (which is also present at the costamere) (Bays et al. 2017; Van Itallie et al. 2014). Dystrophin, while present in smooth muscle is distinctly absent from the dense plaques, but rather localized to caveolae (North et al. 1993). Again there are also structural differences and dense plaques appear in cross sections as discrete foci at the membrane, however in longitudinal sections these structures can extend the entire length of the cell (Small 1985). Because dense plaques are directly coupled to contractile stress-fibres they might be comparable to central rather than peripheral focal adhesions. However, since the dense plaque adhesome has not been analysed yet to our knowledge, it is unclear how much they resemble each other. It is clear however that several key proteins at the dense plaques are regulated in response to forces in similar way as in non-muscle cells. e.g. Force development in smooth muscle cells has been proven to evoke talin and paxillin phosphorylation (Pavalko et al. 1995; Wang et al. 1996), and such phosphorylation requires myosin II activity (Kuo et al. 2011; Lehman and Morgan 2012). Another study on mouse uterus smooth muscle cells, under chronic stretch during pregnancy demonstrated their ability to sense and mechanically adapt to chronic changes in exogenous forces (Wu et al. 2008). This study further found a positive causality between the pregnancy related chronic stretch and the expression level of dense plaque proteins, FAK and paxillin, as well as increases in the phosphorylation of FAK, paxillin, c-Src and ERK1/2. This demonstrates the ability of smooth muscle cells to sense and adapt to mechanical stress by changing the phosphorylation state of adhesion proteins accordingly, leading to enhanced resilience along the smooth muscle cells (Wu et al. 2008). Such findings could further imply a similar regulatory mechanism in vascular smooth muscle cells coping to chronic mechanical stress due to disease conditions such as hypertension and atherosclerosis.

### Podosomes

In addition to dense plaques, vascular smooth muscle cells can form another type of cell–matrix adhesions: podosomes (Fig. 2a, c). Podosomes share a subset of adhesion proteins, scaffolding proteins, signalling proteins, cytoskeleton and the associated regulatory proteins with focal adhesions (Block et al. 2008). Podosomes cons of a dense F-actin core, surrounded by a looser actin meshwork and key actin regulators (Gimona 2008; Joosten et al. 2018; Luxenburg et al. 2007; Tanaka et al. 2015), as well as a surrounding ring of adhesion and scaffolding proteins that connect to a cap structure above the actin core through contractile actomyosin filaments (Burgstaller and Gimona 2005; Cox and Jones 2013; Monypenny et al. 2011; Murphy and Courtneidge 2011). The dense actin core is assembled through ARP2/3, which in turn is activated through Cortactin and WASP in the podosome capping structure (Albiges-Rizo et al. 2009; Destaing et al. 2011; Webb et al. 2006). While Arp2/3 is also the main nucleator for the 2D actin gel of the lamellipodium it indeed has a tendency to form three-dimensional structures when activators are present in certain geometries, as has been demonstrated in vitro by Thery et al. on micropatterns or also found in cyto in certain cell types such as T-cells (Galland et al. 2013; Tabdanov et al. 2015). The adhesion ring again includes a set of key molecules, including integrins, talin, vinculin or filamin (Guiet et al. 2012; Linder et al. 2011; van den Dries et al. 2013). It was suggested that the actomyosin tension in the periphery is allowing the central core to provide the protrusive force that is applied to the surface in a pulsatile manner (Labernadie et al. 2014). Despite this requirement for peripheral actomyosin tension, podosome formation is however promoted by low actomyosin contractility, whereas focal adhesion growth is generally promoted by actomyosin tension (Burridge and Guilluy 2016; Yu et al. 2013), as discussed above. Recent work suggested that this inverse relationship is regulated through microtubules which deliver the RhoGEF, GEF-H1 to the different adhesion sites. Microtubules are connected to talin at podosomes and focal adhesions through a complex of KANK liprins, ELKS and LL5β (Bouchet et al. 2016; Rafiq et al. 2019; Sun et al. 2016). The connection through this complex enables sequestering the RhoGEF, GEF-H1, and hence suppressing actomyosin contractility and promoting podosome formation and/or focal adhesion disassembly. In addition to contractility, also the effect of filamin appears to be inverse for focal adhesions and podosomes. Through inhibition of integrin activity, filamin regulates focal adhesion disassembly (Lynch et al. 2011, 2013; Xu et al. 2010). On the other hand the presence of filamin was reported to be necessary for the formation of podosomes and especially podosome rosettes, although it is not entirely clear how this is reconcilable with its function as integrin inhibitor (Guiet et al. 2012).

Whilst podosomes have the ability to adhere to and degrade extracellular matrix, recent data suggest that they also participate in cellular mechanotransduction (Linder and Wiesner 2016; Parekh and Weaver 2016). Similar to focal adhesions, mechanotransduction by podosomes appears to be bi-directional and the ability to sense the passive forces from a rigid substratum requires podosomes to exert forces onto the substratum. Podosome appearance (Collin et al. 2006; Juin et al. 2013), abundance (Juin et al. 2013; Parekh et al. 2011), and functions in terms of proteolytic ability (Alexander et al. 2008; Parekh et al. 2011) are determined by extracellular stiffnesses. However, whether soft or hard surface is more preferable to podosome formation is debatable and cell type dependent. Vascular smooth muscle cells could form podosome-like protrusions without chemical stimuli on soft 3D matrices (Furmaniak-Kazmierczak et al. 2007) and when placed in a pressure chamber, mimicking stage II hypertension (Kim et al. 2015). In dendritic cells, podosome are preferably formed at pores with lower physical constraint on hard substrate (Baranov et al. 2014). In contrast, endothelial cells form more and larger podosomes on harder stiffnesses (20 kPa) compared to soft stiffness (2 kPa) (Juin et al. 2013). Similarly, in kidney cells, hard surfaces give larger, more persistent, and less dispersed podosomes (Collin et al. 2006). This discrepancy could be due to the differences in the intensities of multiple intracellular signalling pathways, both biochemical and mechanical, which remain elusive and requires further investigation.

Besides extracellular stiffness, extracellular topography is also a determining factor to podosome formation. Apart from stiffness, geometry and topography also matters to the size and duration of podosomes. This appears to be true for smooth muscle as well as non-muscle cells. Vascular smooth muscle cell podosome formation is induced when the cells face microfabricated barriers that mimic the situation of VSMC contacting a stent in vivo (Kim et al. 2015). Dendritic cells reinforce podosome stability on 3D matrices by suppressing Prostaglandin E2 and downstream Rho pathway activity (van den Dries et al. 2012). Miniature rough surface favours large podosome rings in dendritic cells and can extend the podosome lifetime from minutes to hours (Geblinger et al. 2010). As mentioned previously, podosomes also preferably form on soft pores with low physical constraint instead of the surrounding hard substrates (Gawden-Bone et al. 2010). Apart from their mechanosensory properties, podosomes are also mechanical devices exerting forces on the extracellular environment. Importantly, this behaviour is responsive to extracellular stiffnesses as well, since actin polymerization dependent protrusive force generation changes with extracellular stiffness (Collin et al. 2008; Labernadie et al. 2014).

## Conclusions

There is strong evidence for a role of extracellular matrix stiffness in cardiovascular disease and the literature regarding the effects of the mechanical stimuli on cardiomyocytes and vascular smooth muscle cells is expanding rapidly. However, there are still many open questions towards the molecular mechanisms behind rigidity sensing and especially the differences between the various cell and adhesion types. Proteomic analysis of muscle cell adhesions similar to the work from non-muscle cells could help shed light on these differences. Moreover, tools and approaches to analyse the behaviour of different integrins [e.g. DNA origami nanoarrays (Hawkes et al. 2019)] or to analyse cellular or molecular forces with high temporal and spatial resolution (such as nanopillars or tension sensors) will help to better understand the specifics of cardiac or smooth muscle cell mechanosensing.

## Abbreviations

CM: Cardiomyocytes
CF: Cardiac fibroblasts
VSMC: Vascular smooth muscle cells
ECM: Extracellular matrix
CVD: Cardiovascular diseases
MI: Myocardial infarction
HCM: Hypertrophic cardiomyopathy
DCM: Dilated cardiomyopathy
MMP: Matrix metalloproteinases
ROS: Reactive oxygen species
FAK: Focal adhesion kinase
DGC: Dystrophin-glycoprotein complex
nmM: Non-muscle myosin
